# CO-Releasing Molecule (CORM)-3 Ameliorates Spinal Cord-Blood Barrier Disruption Following Injury to the Spinal Cord

**DOI:** 10.3389/fphar.2020.00761

**Published:** 2020-06-04

**Authors:** Gang Zheng, Fanghong Zheng, Zucheng Luo, Haiwei Ma, Dongdong Zheng, Guangheng Xiang, Cong Xu, Yifei Zhou, Yaosen Wu, Naifeng Tian, Yan Wu, Tan Zhang, Wenfei Ni, Sheng Wang, Huazi Xu, Xiaolei Zhang

**Affiliations:** ^1^Department of Orthopaedics, The Second Affiliated Hospital and Yuying Children's Hospital of Wenzhou Medical University, Wenzhou, China; ^2^Zhejiang Provincial Key Laboratory of Orthpaedics, Wenzhou, China; ^3^The Second School of Medicine, Wenzhou Medical University, Wenzhou, China; ^4^Department of Neurosurgery, The Second Affiliated Hospital and Yuying Children's Hospital of Wenzhou Medical University, Wenzhou, China; ^5^Department of Orthopaedics, The Second Affiliated Hospital, School of Medicine, Zhejiang University, Wenzhou, China; ^6^Department of Orthopaedics, Shaoxing People's Hospital, Shaoxing, Wenzhou, China; ^7^Department of Orthopaedics, Chinese Orthopaedic Regenerative Medicine Society, Hangzhou, China

**Keywords:** spinal cord injury, blood spinal cord barrier, carbon monoxide, neutrophil, CO-releasing molecule-3

## Abstract

Spinal cord injury (SCI) is a clinical tough neurological problem without efficient cure currently. Blood-spinal cord barrier (BSCB) interruption is not only a crucial pathological feature for SCI process but is a possible target for future SCI treatments; however, few treatments have been developed to intervene BSCB. In the present study, we intravenously injected CO-releasing molecule3 (CORM-3), a classical exogenous CO donor, to the rats experiencing SCI and assessed its protection on BSCB integrity in rats. Our results demonstrated that the exogenous increasing of CO by CORM-3 blocked the tight junction (TJ) protein degeneration and neutrophils infiltration, subsequently suppressed the BSCB damage and improved the motor recovery after SCI. And we certified that the CO-induced down-regulation of MMP-9 expression and activity in neutrophil might be associated with the NF-κB signaling. Taken together, our study indicates that CO-releasing molecule (CORM)-3 ameliorates BSCB after spinal cord injury.

**Graphical Abstract:**
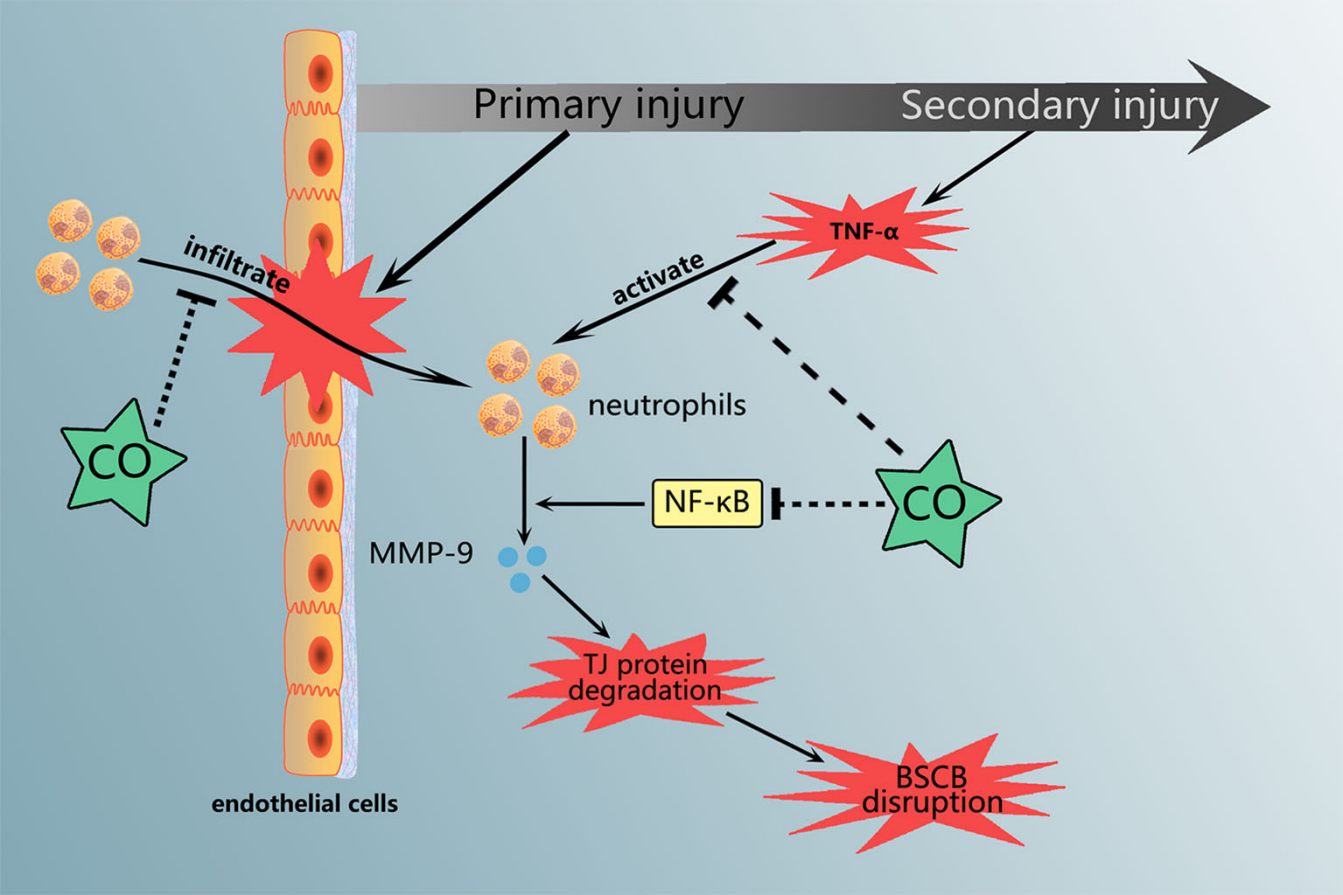
Carbon monoxide treatment attenuated the neutrophils infiltration and MMP-9 expression and activation, which are associated with NF-κB pathway.

## Introduction

Spinal cord injury (SCI) causes longstanding physical and sensory impairment and can be fatal. Traumatic SCI consists of two stages: (1) the primary injury refers to the immediate local mechanical impact and (2) the inferior injury is identified as a multifaceted cascade representing for local edema, hemorrhage, oxidative stress, inflammation, and apoptosis (Penas et al., 2010). Alleviating and delaying the secondary injury process is identified as the potential target for SCI treatment (Giszter, 2008). But previous studies principally focus on neuronal death and regeneration. The effect of blood spinal cord barrier (BSCB) for this process has yet to be sufficiently investigated (Fassbender et al., 2011; Sharma, 2011).

Under normal physiological condition, the BSCB could prevent the entry of pathogens, blood-derived products, and cells into the spinal cord to maintain homeostasis within the spinal cord (Bartanusz et al., 2011; Kumar et al., 2016). The barrier function bases on non-honeycomb shaped endothelial cells (ECs) and their additional constituents such as pericytes and astrocytic end feet (Palmer, 2010). ECs in the central nervous system (CNS) characterize by plentiful cytosolic mitochondria and the absence of cell membrane fenestrations and pinocytic vacuoles (Bartanusz et al., 2011). Meanwhile, ECs produce tight junction (TJ) proteins to form the complicated network, which restricts the paracellular diffusion pathway between the ECs. These structural features ensure the formation of stable capillary microenvironment (Obermeier et al., 2013). Unfortunately, primary injury causes irreversibly local BSCB disruption, which allows the inflammatory cells (primarily neutrophils) infiltrate and migrate into the uninjured section and subsequently exacerbates the secondary sequential damage by secreting neurotoxic factors such as various matrix metallopeptidases (MMPs), reactive oxygen species (ROS), and inflammatory cytokines (Sharma, 2011; Kumar et al., 2016). The abnormal expression and activation of MMPs, especially MMP-9 could promote the TJ proteins degeneration, the fundamental molecular components of BSCB (Yu et al., 2008; Lee et al., 2012). The genetic or pharmacological suppression of MMP expression and activation or direct blocking the neutrophil infiltration have been demonstrated the protection of BSCB following SCI (Noble et al., 2002; Wells et al., 2003; Yu et al., 2008; Kumar et al., 2016).

Heme oxygenase (HO) catalyzes heme degradation with the CO production, which has been proved with biological function including anti-inflammation, anti-apoptosis, and anti-oxidative at low dose (Kim et al., 2007; Motterlini and Otterbein, 2010; Wang et al., 2010). In 2009, Zeynalov and Dore found CO inhalation reduced infarct volume and attenuated brain edema following mouse ischemic brain injury (Zeynalov and Doré, 2009). Most recently, Choi et al. reported that the CO-releasing molecule (CORM)-3 treatment, the exogenous CO donor, ameliorated the prognosis of traumatic brain injury (TBI) by promoting neurogenesis through the rescuing the crosstalk between the pericyte and neural stem cell (Choi et al., 2016). Nevertheless, the studies about CO effect in SCI mainly focus on the HO. Applying hemin, chemicals, and natural products to regulate HO expression or activity subsequently inducing the CO production, which not have enough evidence to confirm the CO effect for BSCB (Lin et al., 2016; Lin et al., 2017; Lu et al., 2018; Wei et al., 2018; Zheng et al., 2019). Therefore, we performed this research to make up for this question.

Recently, our group reported that increasing CO by CORM-3 alleviated neurological deficits and improved the functional recovery after SCI. And we detected CO level and HO-1 expression variation within 7 days following SCI (Zheng et al., 2019). In this article, we assessed the protection of CO in BSCB destruction. And CORM-3 treatment attenuated the neutrophils infiltration and MMP-9 expression and activation *in vivo* and *in vitro*. And we initially examined the potential mechanism for CO-regulated MMP-9 production in neutrophil, which might be associated with NF-κB pathway.

## Methodology and Materials

### Antibodies and Chemical Reagents

Carbon monoxide releasing molecule 3 (CORM-3) were obtained from Sigma-Aldrich (St. Louis, MO, USA). Antibody against ZO-1 and Occludin were bought at Santa Cruz Biotechnology (Santa Cruz, CA, USA). Antibody against Ly6G, p-p65, p65, GAPDH, MMP-2/-3/-9, and Lamin B were bought at Abcam (Cambridge, CA, USA). TNF-α and anti-ICAM-1 were purchased from R&D system (Minneapolis, MN, USA). Anti-MPO was bought at Bioworld (Minneapolis, MN, USA)

### The SCI and Animal Model

The Sprague–Dawley female adult rats (220–250 g, 2 months old) were bought at the Animal Center of the Chinese Academy of Sciences in Shanghai, China. The ethical procedures for handling the rats referred to the guidelines and permission from the Animal Care and Use Committee of Wenzhou Medical University (ethic code: wydw2014-0129).

The rats were randomized into four groups and they were given intraperitoneal sodium pentobarbital injections (65 mg/kg) for sedation. After their skins were prepared and sterilized at the back, skin incisions were done lengthwise on the dorsal midline to reveal the vertebral column and they underwent surgical removal of the lamina at the T9 level. The visible spinal cord underwent a simulated crush injury by compressing it with a vascular clasp (30 g force; Oscar, China) for a minute. Surgery consisting of a laminectomy at level T9 without exposure to spinal crush injury, was also performed in the rats of Sham group. The post-surgical care consisted of manually emptying the urinary bladder two times a day the normal bladder function returned and treatments consisting of cefazolin sodium (50 mg/kg). The rats that experienced SCI were randomized into three groups; group treatments included CORM-3/iCORM-3/saline. To investigate the effects of CO, preparations of inactive CORM-3 (iCORM-3) were made by placing CORM-3 in saline (pH = 7.4) overnight at room temperature to complete the CO releasing process (Zhang W. et al., 2017). The CORM-3 underwent dilutions involving normal saline and completed an ultimate CORM-3 concentration of 8 mg/ml. The CORM-3 solution was instantly injected to the rat tail veins after surgery with a dosage of 8 mg/kg per day until they were euthanized. Corresponding quantities of iCORM-3 and normal saline were injected for vehicle regulation.

### Examination Using Evans Blue Dye

The BSCB permeability was detected by Evans blue dye as previously described (Zhang D. et al., 2017). Three days after injury to spinal cord, the rats underwent injections of 2% Evans blue dye (2 ml/kg) mixed in saline solution intravenously *via* the veins of the tail section. After 2 h, rats were euthanized with sodium pentobarbital (65 mg/kg, i.p.), then underwent perfusion in 0.9% normal saline. The lesioned spinal cord EB tissues were quantified and absorbed in N,N'-dimethylformamide (Jinsan, Wenzhou, China) at 50°C for 72 h. The supernatant's optical density was investigated using an enzyme-labeled meter (at an excitation wavelength of 620 nm and an emission wavelength of 680 nm). The presence of the dye in samples indicated existence of lg/g. After being injected with EB, the rats were subjected to fixation *via* perfusion with 4% paraformaldehyde for 2 h. The frozen section machine cut the spinal cord tissues into segments of 10 μm thickness at 20°C, then the segments underwent observation using a fluorescent microscope (Olympus, Tokyo, Japan).

### Gelatin Zymography

Gelatin zymography was done as mentioned earlier. The MMP-2,9 activity *in vivo* at day 3 after SCI was detected by Gelatin Zymography. The core of lesioned spinal cord (0.5 cm in length) was standardized in a lysis buffer consisting of 50 mM Tris-HCl, pH 8.0, 150 mM NaCl, 1% NP-40, 0.5% deoxycholate, and 0.1%SDS. Equal protein quantities (40 ug) were put on 10% SDS-polyacrylamide gel electrophoresis, co-polymerized with 1 mg/ml gelatin (Sigma-Aldrich). The gel was subjected to washing in 2.5% Triton X-100 after electrophoresis for 30 min and underwent incubation for 24 h at 37°C in substrate buffer, together with Tris 50 mM/l (pH 7.6), CaCl 25 mM, NaCl 0. 2mM, and 0.02% (w/v) Brij-35 (Sigma-Aldrich). Finally, the Coomassie blue solution stained the gel for 4 h and underwent de-staining with 10% acetic acid/40% methanol. The gels underwent scanning for quantitative examination and the positive band was quantified by NIH Image J software (NIH). *In vitro*, MMP-9 in regulated media was investigated using gelatin zymography as mentioned earlier.

### Human Brain Microvascular Endothelial Cells Culturing and Treatment

Human brain microvascular endothelial cells (HBMECs) and endothelial cell medium were bought at ScienCell Research Laboratories (ScienCell Research Laboratories, San Diego, CA, USA). Cells were incubated in a moistened environment consisting of 5% CO2 and 95% air at 37°C. Cells were pretreated for 6 h with CORM-3 (100 μm).

### Primary Neutrophil Culturing and Treatment

The Percoll gradient was laminated with SD adult rat's (200–250 g) blood in layer densities of 45, 54, and 63%. To detect the neutrophils between the second and third layer, the blood was prepared using gradient centrifuge (3,000 rpm for 15 min). The erythrocyte lysis and ACK buffer purified the cells. The granulocytes underwent washing in HBSS buffer and diluting in RPMI1640 media with 10% (v/v) heat-inactivated FBS followed by plating the neutrophils in the six-well dish with a density of 100,000 cells/cm^2^ (Pashevin et al., 2015). The TNF-α (100 ng/ml) and CORM-3 (100 μm) were used to treat the neutrophils for 6 h.

### Co-Culture of HBMECs and Neutrophils

One part of HBMECs were pretreat with CORM-3 (100 μm) for 6 h and then washed out followed by addition of neutrophils to the HBMECs cultures at a concentration of 1 × 10^6^ cells per ml. In addition, one part of these neutrophils had been pretreated with TNF-a (100 ng/ml) for 6 h. The unstimulated as well as the activated neutrophils were harvested, washed, and then added to the HBMECs cultures as concentrate. The HBMEC-neutrophil co-culture were maintained at 37°C for 6 h and then neutrophils were washed out. Finally, HBMECs were collected and processed for western blotting analyses and immunofluorescence staining.

### Western Blot Assay

RIPA lysis buffer consisting of 1 mM PMSF extracted whole proteins then their concentrations were quantified by the Enhanced BCA Protein Assay Kit (both from Beyotime, Shanghai, China) *via* a Microplate Reader (Molecular Devices Flexstation 3, USA). A protein tissue of 40 ng was parted by sodium dodecylsulfate-polyacrylamide gel electrophoresis (SDS PAGE) and transported to a polyvinylidene diﬂuoride membrane (Bio-Rad, California, USA). The 5% nonfat milk was used for blocking process for 2 h, then the membranes underwent incubation with the primary antibodies against ZO-1 (1:500), Occludin (1:500), ICAM-1 (1:1,000), MPO (1:500), MMP-2/-3 and -9 (1:1,000 respectively), p65 (1:500), Lamin B (1:1,000), GAPDH (1:5,000). The membranes underwent washing with TBS thrice for 5 min, followed by treatment in horseradish peroxidase-conjugated secondary antibodies. After washing thrice in TBST, the visualization of the blots was done *via* electrochemiluminescence plus reagent (Invitrogen, Carlsbad, USA). In the final phase, the Image Lab 3.0 software measured the blot intensities (Bio-Rad, California, USA).

### Immunofluorescent Staining

Three days after injury to the spinal cord, the tissue samples were collected. After harvesting, the spinal cord segments were fixed in 4% PFA, followed by washing and then embedded in paraffin. The transverse segments of 5 μm thickness underwent cutting, deparaffining in xylene, and rehydration in ethanol rinses. The segments underwent incubation in 10% normal goat serum for 1 h at room temperature in PBS consisting of 0.1% Triton X-100. This was followed by incubation using suitable primary antibodies overnight at 4°C in a similar buffer. Based on their varying targets, primary antibodies that were used included: Ly6G (1:100) and MMP-9 (1:100). After incubating the primary antibodies, segments underwent washing for 40 min, followed by incubation with Alexa Fluor 488/594 goat anti-rabbit/mouse secondary antibodies at room temperature for 1 h. The sections underwent rinsing thrice with PBS and incubation with 4,6-diamidino-2-phenylindole (DAPI) for 10 min, followed by washing in PBS and a coverslip sealed them. The fluorescent microscope (Olympus Inc., Tokyo, Japan) recorded the images, then three investigators quantified the positive neurons in each segment while being blinded to the groups under investigation. The values from the counting of 30–40 random sections produced the calculations for the rates of corresponding-protein positive cells per section examining five rats each group.

### Statistical Analysis

The outcomes were represented by mean ± S.D. The SPSS statistical software program 20.0 (IBM, Armonk, NY, USA) analyzed the data. The one-way analysis of variance (ANOVA) and Tukey's test were used to compare the experimental groups. The BBB scores underwent analysis using Mann–Whitney test. Statistical values of *P* < 0.05 were stated as significant.

## Results

### Carbon Monoxide Attenuates BSCB Disruption and TJ Protein Loss Following SCI

Recently, we have demonstrated CORM-3 treatment alleviated neurological deficits and improved the functional recovery after SCI *via* increasing the CO content in spinal cord (Zheng et al., 2019). In this article, we mainly discussed CO protection in BSCB function after SCI.

**Figure 1:**
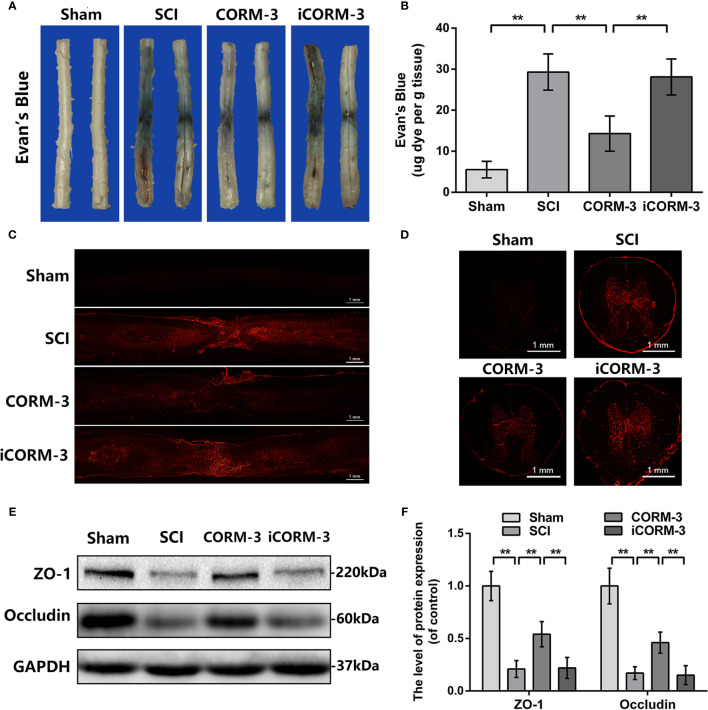
Carbon monoxide inhibits BSCB interruption and declined TJ proteins at 3 days following SCI. **(A)** Characteristic complete spinal cords show that Evan's Blue dye infused into spinal cord at 3 days. **(B)** Quantifying the Evan's Blue at 3 days (μg/g). **(C, D)** Characteristic fluorescent images of Evans Blue Dye extravasation at 3 days. (bars: 1 mm). **(E, F)** The protein expression of ZO-1 and Occludin in spinal cord at 3 days post-SCI. All data is denoted as mean ± S.D. (n = 5). **P < 0.01.

Evan's Blue dye to test BSCB permeability *via* tail vein at 3 days following SCI. By the Evan's Blue staining, less blue dye appears around the damaged area in CORM-3 group relative to the SCI and iCORM-3 groups as well as the quality results in **Figures 1A, B**. Meanwhile, compared to the SCI and iCORM-3 groups, CORM-3 treatment weakened the red fluorescence intensity in the transverse and longitudinal section of spinal cord (**Figures 1C, D**). As the fundamental molecular structure of BSCB, SCI reduces ZO-1, and Occludin protein expression, whereas CORM-3 reversed this trend (**Figures 1E, F**).

### CO Reduces the MMP-9 Production and Blocks Neutrophil Infiltration After SCI

**Figure 2:**
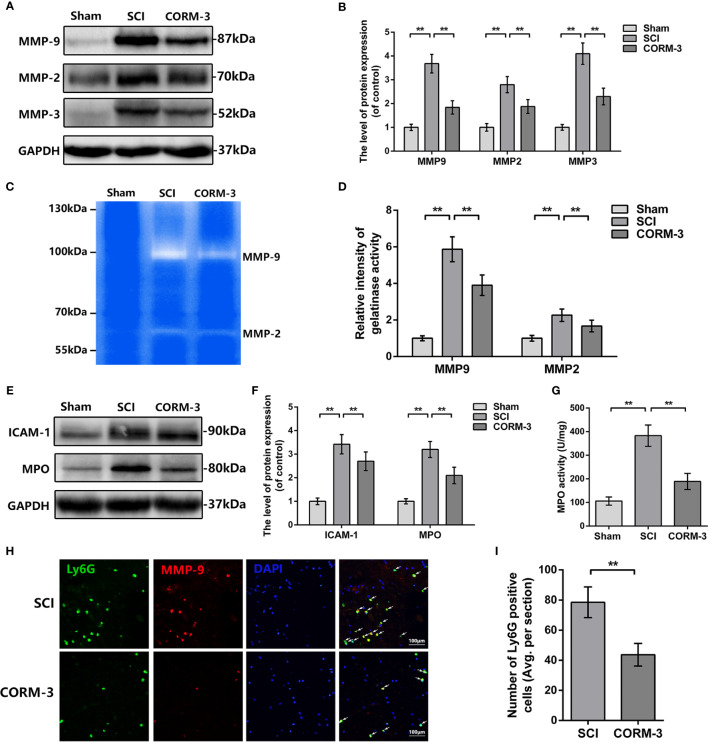
CORM-3 blocks the neutrophil infiltration and inhibits MMP-9 production and activation at 3 days after SCI. **(A, B)** The protein expression of ICAM-1 and MPO in spinal cord at 3 days post-SCI. **(C)** MPO activity in spinal cord at 3 days post-SCI. **(D, E)** The protein expression of MMP-2, -3, and -9 in spinal cord at 3 days post-SCI. **(F, G)** Dual immunofluorescence of MMP-9 and Ly6G in segments from tissues at 3 days post-SCI (bar: 50 μm). The white arrow indicates the Ly6G/MMP-9 positive cells. **(H, I)** Representative zymography and measurement data of MMP-2 and -9 in spinal cord at 3 days post-SCI. All data is denoted as mean ± S.D. (n = 5). **P < 0.01.

During the SCI, the abnormal increase of MMPs production and activity contribute to TJ protein degradation (Lee et al., 2014; Kumar et al., 2017; Wang et al., 2017). By western blot assay, SCI-induced upregulation of MMP-2, -3, and -9 expression was significantly reduced by CORM-3 treatment (**Figures 2A, B**). And the gelatin zymography results revealed the CORM-3 obviously suppressed the activation of MMP-2 and -9 (**Figures 2C, D**).

As the primary source of MMP-9 in the early stage post-SCI, infiltrating neutrophil was measured by western blot and immunofluorescence (de Castro et al., 2000; Neirinckx et al., 2014). As shown in **Figures 2E, F**, the expression of intercellular adhesive molecule 1 (ICAM-1), linked to leukocyte adhesion, and Myeloperoxidase (MPO), a peroxidase enzyme primarily expressing in neutrophils and storing in azurophilic granules were increased after SCI, whereas CORM-3 treatment alleviated these phenomena (Neirinckx et al., 2014; Patterson et al., 2014). The outcomes of MPO activity assay were similar to the western blot (**Figure 2G**). To determine the infiltration levels of neutrophils following SCI, we performed a double-labeling immunofluorescence staining with MMP-9 and Ly6G (a neutrophil marker). As shown in **Figures 2H, I**, CORM-3 injection decreased the number of Ly6G-positive cells on 3 days post-SCI. Furthermore, we can obviously discovered that the MMP-9 and Ly6G fluorescent could basically be collocated, which again answers the source of the MMP-9.

### Regulation of MMP-9 Expression and Activation by CO in Neutrophils Is Related to *In Vitro* NF-κB Signaling Pathway

**Figure 3:**
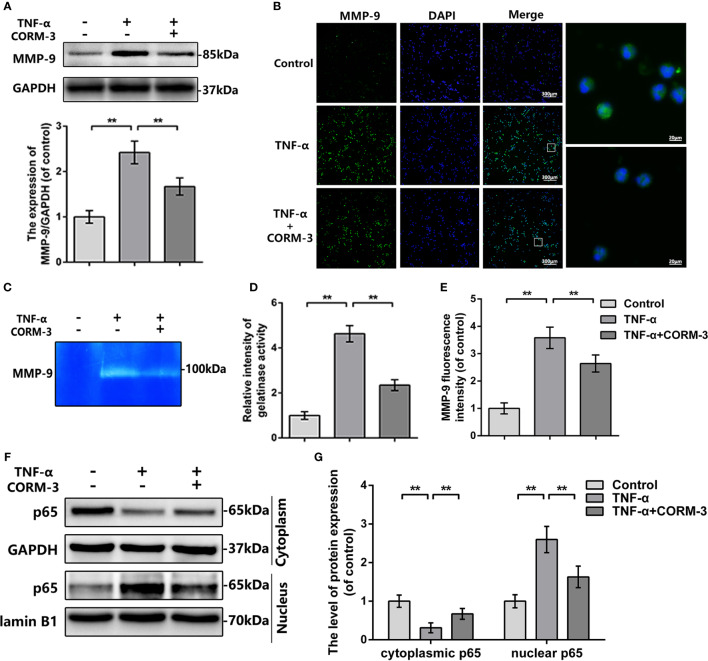
MMP-9 regulation of CORM-3 is related to NF-κB signaling pathway in neutrophils *in vitro*. **(A)** The protein expression of MMP-9 in neurons were treated as above. **(B)** The immunofluorescence of MMP-9 in the neutrophils. (bars: 300μm or 20μm). **(C, D)** Representative zymography and measurement data of MMP-2 and -9 in neutrophils treated as above. **(E)** Quantification of the fluorescence intensity of MMP-9 in neutrophils treated as above. **(F, G)** The protein expression of p65 in neutrophils treated as above. All data is denoted as mean ± S.D. (n = 5). **P < 0.01.

To investigate the mechanism of CORM-3-medicated MMP-9 regulation, we extracted neutrophils from blood and stimulated with TNF-α to imitate the SCI model *in vitro*. The western blot, gelatin zymography, and MMP-9 immunostaining outcomes illustrated that CORM-3 inhibited TNF-α-stimulated MMP-9 expression and activate neutrophils, which are consistent with our *in vivo* results (**Figures 3A–E**).

In neutrophils, NF-κB signaling is essential in MMP-9 synthesis and activation (Tahanian et al., 2011). TNF-α causes the p65 transfer to the nucleus and next initiate transcription and translation of MMP-9 (Dilshara et al., 2015). We investigated the levels of p65 expression in the cytoplasmic and nuclear portions of neutrophils. After TNF-α stimulation, there were elevated nuclear p65 expressions then the cytoplasmic p65 were declined, whilst CORM-3 treatment inhibited this response (**Figures 3F, G**). These data indicated that NF-κB pathway could be considered as the potential mechanism of CORM-3 protection. Additionally, our *in vivo* experiments also showed that CORM-3 treatment inhibited NF-κB pathway activation after SCI (**Figure S1**).

### CORM-3 Alleviates TJ Protein Degradation Induced by Activated Neutrophils in HMBECs *In Vitro*

**Figure 4:**
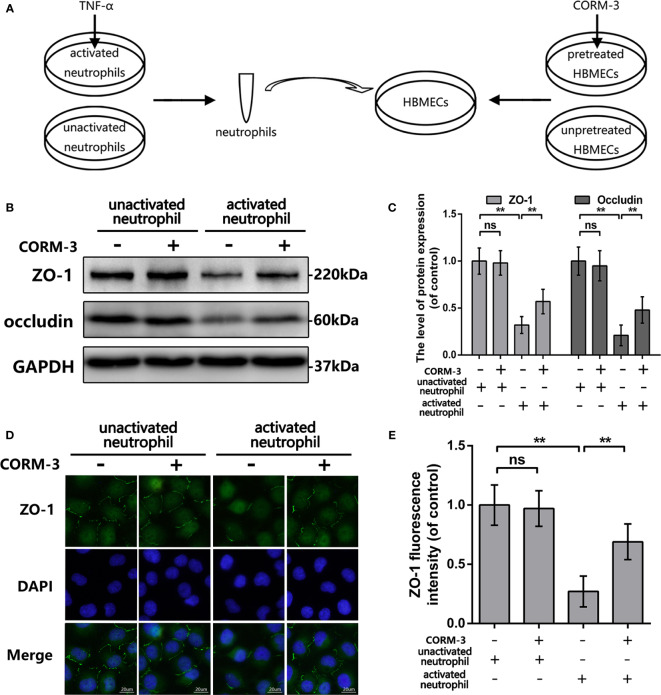
CORM-3 alleviated TJ protein degradation induced by activated neutrophils in HMBECs, *in vitro*. **(A)** Schematic experimental settings for the neutrophils and HBMECs co-culture. **(B, C)** The protein expression of ZO-1 and Occludin in HBMECs treat as above. **(D, E)** Representative immunofluorescence staining and quantification data of ZO-1 in HBMEC. (bar: 20 μm). All data is denoted as mean ± S.D. (n = 5). **P < 0.01. NS, No significant.

To explain the CO-induced protection, we pretreated HBMECs by CORM-3 and then co-cultured with neutrophils. In our experimental settings (**Figure 4A**), we used TNF-α to induce neutrophils activation and removed after 6 h incubation. Neutrophils were harvested, washed, and added to HBMEC as concentrate. Before adding neutrophils, one part of HBMECs was pretreated with CORM-3. As shown in **Figures 4B, C**, activated neutrophils caused prominent loss of ZO-1 and Occludin in HBMECs, whereas CORM-3 pretreatment in HBMEC reversed it. Interestingly, CORM-3 did not influence the ZO-1 and Occludin expression in HBMEC under inactivated neutrophils co-culture conditions. These results are consistent with our immunofluorescence staining (**Figures 4D, E**).

## Discussion

Although cell transplantation, drug and growth factor applications could reduce BSCB damage, the therapeutic efficacy also be affected as the restore of BSCB function (Kumar et al., 2016). Compare to the above treatments, carbon monoxide, as a gas molecule, could pass through the BSCB and reach the spinal segments, which are far from the primary damaged area.

As one of the degradation products of heme, CO has been proved with biological function including anti-inflammation, anti-apoptosis, and anti-oxidative at low dose (Motterlini and Otterbein, 2010; Choi et al., 2016). Several animal experiments proved that providing CO through inhaling or exogenic CO donors, the CORM-3, alleviates neural inflammation, neuron death and promotes neurogenesis (Zeynalov and Doré, 2009; Choi et al., 2016; Wang et al., 2018). However, to our knowledge, the effect of CO in BSCB protection after SCI is still unknown.

Following SCI, local mechanical impact to the spinal cord directly results in the vascular rupture and tissue destruction and consequently enhances the heme synthesis (extracted from hemoglobin/dying cells) (Gozzelino et al., 2010; Grochot-Przeczek et al., 2012). Simultaneously, HO-1was raised and activated compare to the uninjured spinal cord (Queiroga et al., 2015). These underlining mechanisms explains the SCI-initiated CO content variations in the spinal cord.

Normally, the BSCB blocks the entry of toxic substances into the spinal cord microenvironment. Succeeding acute spinal cord trauma, the BSCB disruption is defined as the fatal event which contributes to the secondary-injury and characterizes with the time dependence that initiates within 5 min, peaks at 24 h, and lasts for up to 28 days or longer and extends to the local domain of the spinal cord (Noble and Wrathall, 1989; Runge et al., 1997). Due to the barrier opening and chemokines secretion, the neutrophils infiltration further exacerbates BSCB damage by producing various matrix metallopeptidases (MMPs), especially MMP-9. And, the overproduction of MMP-9 facilitates the degeneration of TJ protein and basal lamina. In the mouse model of ischemic stroke, the CORM-3 administration by retro-orbital injection alleviated the BBB disruption and neurologic deficit in rat stroke model (Wang et al., 2018). However, whether CO protect BSCB following SCI remains to be studied. Our results demonstrated the BSCB permeation, neutrophils infiltration, TJ protein degeneration, and MMP-9 activity in spinal cord are increased after injury, but CO treatment reversed these phenomena. Previous studies reported carbon monoxide could regulate the ICAM expression, which is an adhesion molecule that is constitutively expressed in ECs at low levels but exhibits increased levels of expression under hypoxic conditions (Hopkins et al., 2004). Lee et al. reported that CORM-2 attenuates ICAM-1 expression in *P. aeruginosa*-induced lung inflammation mice and human pulmonary alveolar epithelial cells (Yeh et al., 2014). Adhesion molecules generally encoded by the transcription factor such as NF-κB. In the TNF-α and IL-1β exposed human gingival fibroblasts, CORM-3 inhibited adhesion molecules expression by suppressing the NF-κB pathway (Song et al., 2011). Additionally, Cunha and his colleagues demonstrated that the CO donor, dimanganese decacarbonyl, sustained the neutrophils adhesion and migration and ICAM-1 expression depending on soluble guanylate cyclase (sGC) activation (Lee et al., 2018). By the isolating the neutrophils from the PKG-deficient mice, they found the HO/CO/PKG pathway involves in neutrophil migration which might be further explain by the PKG effect on the NF-κB phosphorylation. However, Stirling et al. reported that inhibiting neutrophils by anti-Ly6G/Gr-1 aggravated the neurological outcome, which seems to be contrary to our results. This discrepancy results may be caused by the number of remaining neutrophils (Stirling et al., 2009). The use of an anti-Ly6G/Gr-1 antibody could abolish subtotal systemic neutrophils whereas CORM-3 acts by decreasing the neutrophils at the site of SCI.

Compare to wild-type mice, MMP-9 knockout mice exhibited the more BSCB integrity, the alleviation of neuroinflammation and significant locomotor recovery (Noble et al., 2002). Wang and his colleagues found that the MMP-9 expression and its colocalization level with pericytes were declined by the CORM-3 in the mouse stroke model (Wang et al., 2018). Similarly, our western blot and gelatin zymography results demonstrated that MMP-9 expressions and activities decreased after CORM-3 treatment *in vivo* and *in vitro*. Various studies investigated the NF-κB could bind to the MMP-9 corresponding sequence and control its transcription (Mi et al., 2018; Muscella et al., 2018). In our *in vitro* researches, CORM-3 sustained TNF-α stimulated the p65 nuclear transcription in neutrophils. But the carbon monoxide-induced the NF-κB regulation still needs to further explore. TNF-α activates IκB kinase (IKK) and degrades IκBα, resulting in the liberation and translocation of p65, which then increases transcription of downstream mediators such as MMP-9 and ICAM-1. However, Wung and his colleagues showed that applying CO donors in ECs blocked the TNF-α-induced p65 nuclear translocation by intervening its glutathionylation, but not the IκBα degradation (Yeh et al., 2014). And the CO donors-caused intracellular ROS level slight increase and Nrf2 nuclear accumulation play the important roles in p65 glutathionylation. In brief, the low-level ROS enhances the oxidation to reduction ratio of glutathione (GSSG/GSH) and Nrf2 activation mediated the expression of γ-glutamylcysteine synthetase, which is the rate-limiting enzyme in GSH synthesis (Yeh et al., 2014). These events medicated by CO administration contribute to proteins glutathionylation. The mechanism about CO-induced p65 inactivation in TNF-α exposed neutrophil requires investigation in detail.

Due to CO could competitively bind to hemoglobin with oxygen, it is considered as a toxic gas (Motterlini and Otterbein, 2010). Although CO has multiple biological activities at low concentrations, its therapeutic application is hampered by the lack of a safe and effective delivery (Queiroga et al., 2015). Continuous CO inhalation could rapidly elevate carboxyhemoglobin (COHb) to toxic levels (> 15%) (Otterbein et al., 2000). In our study, we did not detect COHb level in CORM-3 treated rats, but Prabhu et al. reported that the COHb level was stably maintained at 6% during the 24 days medication period in the mice administrating the CORM-3 (40 mg/kg/d) by intraperitoneal injection (Chen, 2014).

## Data Availability Statement

The datasets generated for this study are available on request to the corresponding authors.

## Ethics Statement

The animal study was reviewed and approved by Animal Care and Use Committee of Wenzhou Medical University.

## Author Contributions

GZ wrote the paper, performed the experiments, and generated data. GZ, FZ, and ZL performed the experiments and generated data. YW, NT, and DZ analyzed data. GX, CX, and YZ contributed reagents and materials tools. HX and XZ conceived and designed the experiments. TZ, SW, YSW designed the experiments and helped write the manuscript. HM and WN revised article and provided data.

## Conflict of Interest

The authors declare that the research was conducted in the absence of any commercial or financial relationships that could be construed as a potential conflict of interest.

## Supplementary Material

Figure S1 CORM-3 inhibits NF-κB signaling activity after SCI. (A, B) The protein expression of p-p65 and p65 in spinal cord at 3 days post-SCI. All data is denoted as mean ± S.D.(n=5). ***P* < 0.01.
