# Supplementary figures and images for: CO-Releasing Molecule (CORM)-3 Ameliorates Spinal Cord-Blood Barrier Disruption Following Injury to the Spinal Cord
Source: Front Pharmacol. 2020 Jun 4;11:761. doi: 10.3389/fphar.2020.00761 (PMC7287126; doi:10.3389/fphar.2020.00761)

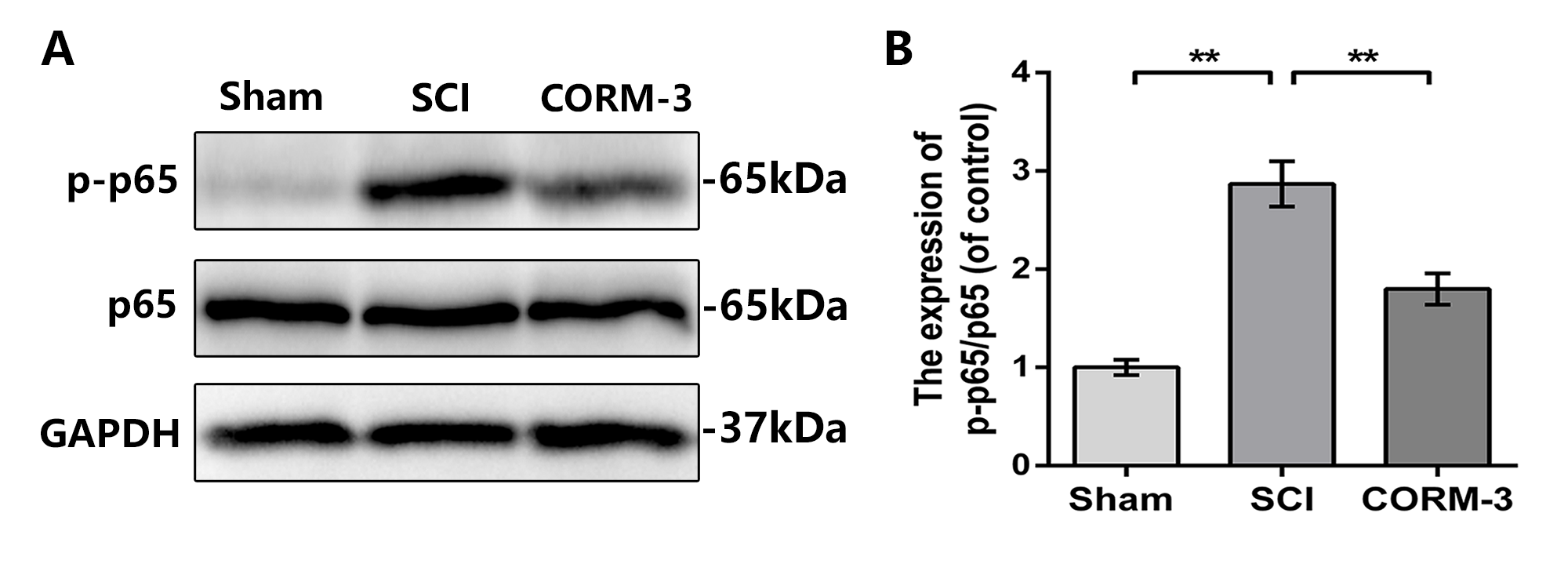

Supplement: Figure S1 — CORM-3 inhibits NF-κB signaling activity after SCI. (A, B) The protein expression of p-p65 and p65 in spinal cord at 3 days post-SCI. All data is denoted as mean ± S.D.(n=5). **P < 0.01. [file Image_1.tif]
